# Factors Associated With HIV-Related Stigma Toward People Living With HIV Among Nurses in Liangshan Yi Autonomous Prefecture, China: A Cross-Sectional Study

**DOI:** 10.3389/fpsyt.2021.714597

**Published:** 2021-08-23

**Authors:** Yao Yin, Angela Chia-Chen Chen, Shaoping Wan, Hong Chen

**Affiliations:** ^1^Department of Neurology/West China School of Nursing, West China Hospital, Sichuan University, Chengdu, China; ^2^College of Nursing and Health Innovation, Arizona State University, Phoenix, AZ, United States; ^3^School of Medicine, Sichuan Cancer Center, Sichuan Cancer Hospital and Institute, University of Electronic Science and Technology of China, Chengdu, China; ^4^West China School of Nursing and Department of Nursing, West China Hospital, Sichuan University, Chengdu, China

**Keywords:** HIV-related stigma, HIV knowledge, HIV/AIDS & infectious diseases, nurses, China

## Abstract

**Background:** The Liangshan Yi Autonomous Prefecture has one of the most serious human immunodeficiency virus (HIV) epidemics in China. Evidence shows HIV-related stigma toward people living with HIV (PLWH) among nurses impedes HIV prevention and treatment. However, only limited research about HIV-related stigma toward PLWH from the perspective of nurses in Liangshan has been conducted.

**Objective:** This study aimed to assess HIV-related stigma toward PLWH among nurses and determine factors associated with it in Liangshan, China.

**Design:** We conducted a cross-sectional survey using a stratified, random cluster sampling method.

**Participants:** Registered nurses (*N* = 1,248; primary hospitals = 102, secondary hospitals = 592, tertiary hospitals = 554) who were aged 18 or older, worked in the selected hospitals for at least 6 months, and consented to participate were recruited.

**Methods:** All participants completed an anonymous online survey measuring sociodemographic characteristics, HIV-related stigma and HIV knowledge. We used multiple stepwise regression analysis to examine factors associated with HIV-related stigma toward PLWH among these nurses.

**Results:** The mean score of HIV-related stigma among nurses was 50.7 (SD = 8.3; range 25–78). Nurses who were more experienced, had higher levels of education, and were working in tertiary hospitals reported higher level of HIV-related stigma. Those who had better HIV knowledge, reported a willingness to receive HIV-related training, were working in areas that had a high prevalence of HIV, had prior experience working in AIDS specialized hospitals, and worked in hospitals that had policies to protect PLWH showed a lower level of HIV-related stigma toward PLWH.

**Conclusions:** Our findings suggested that providing culturally congruent education and training about HIV and care, and having hospitals that promoted policies protecting PLWH, may reduce HIV-related stigma toward PLWH among nurses in China.

## Background

Stigma related to the human immunodeficiency virus (HIV) is prevalent and has been identified to be a major barrier for HIV prevention and treatment (1). Researchers suggest that HIV-related stigma is also still a barrier for people living with HIV (PLWH) to disclose their HIV status and to seek and adhere to treatments; consequently, this barrier has an adverse impact on their health (2–4). HIV-related stigma exists in nursing care worldwide and has large impact on the health outcomes of PLWH (5–9).

Herek (10) defined HIV-related stigma as “prejudice, discounting, discrediting, and discrimination directed at people perceived to have acquired immune deficiency syndrome (AIDS) or HIV” (p. 1107). Manifestations of HIV-related stigma within the healthcare system include neglecting and avoidance of nursing care, exaggerated precautions, judgmental remarks and behavior, labeling patients and viewing them as attention seekers and breaches of confidentiality (11–14). Empirical evidence shows that the main causes of HIV-related stigma among healthcare providers are their lack of awareness of the stigma perceived by PLWH and the negative impact of stigma on PLWH, fear of casual contact due to poor knowledge about HIV transmission and the association of HIV with improper or immoral behavior (15).

Researchers have found that certain sociodemographic characteristics of nurses, such as age, gender, race, marital status, educational level, and certain religious belief are associated with HIV-related stigma toward PLWH (16–21). Furthermore, longer working experience, lower levels of HIV knowledge and nurses working at Islamic hospitals have also been associated with higher levels of stigmatizing attitudes (5, 17, 20, 22).

Findings about the relationship of working at HIV clinics with the level of HIV-related stigma among healthcare providers are mixed in the literature (11, 19, 23). Structural and policy support in HIV clinics have been found to reduce HIV-related stigma toward PLWH in healthcare providers, including HIV-related training, resources for universal precaution and available access to post-exposure prophylaxis (11, 15).

Due to the rapid increase of HIV epidemic in China, Chinese government announced a national AIDS control policy—“Four Frees and One Care”—in 2003. This national policy refers to free antiretroviral drugs treatment to AIDS patients living in rural areas or individuals without insurance living in urban areas, free voluntary counseling and testing (VCT), free drugs treatment to HIV-infected pregnant women to prevent mother-to-child transmission (PMCT) and HIV testing of newborns, free schooling for AIDS orphans, and care and financial assistance to PLWH (24). Under this national policy, a growing number of PLWH have received free tests, consultations, and treatments, and the associated mortality rate has been reduced (24).

In China, the Liangshan Yi Autonomous Prefecture is unique; it has the largest concentration of Yi (彝 族), one of the largest minority groups in China, and has one of the most serious epidemics of HIV in China (25). In fact, nearly 50% of PLWH in Liangshan were infected through injecting drugs (26). Its special cultural background, extreme poverty, low level of education, and prevalent substance use through shared needles in this geographic area have made Yi people particularly vulnerable to HIV infection (27). In order to provide the best possible and culturally congruent care for PLWH, it is critical to optimize nurses' roles in providing HIV-related care (28). However, there is a very little understanding of nurses' HIV-related stigma toward PLWH and factors associated with the stigma in Liangshan. To address this gap, our study aimed to answer the following research questions: (a) what is the status of HIV-related stigma toward PLWH among nurses in Liangshan, and (b) what are the factors associated with HIV-related stigma toward PLWH in nurses in Liangshan?

## Materials and Methods

### Study Design, Setting, and Participants

We conducted a cross-sectional study from September to November 2017 in Liangshan. Hospitals in China are designated as primary, secondary and tertiary hospitals based on a hospital's capacity of providing medical care, education and research. Primary hospitals have <100 beds and focus on health education, disease prevention and health care, rehabilitation, family planning, and common and frequent disease management. Secondary hospitals usually have beds between 100 and 500 and provide comprehensive health care, medical education and research. Tertiary hospitals typically have more than 500 beds and provide comprehensive health services at the city, provincial or national level and carry a more important role in medical education and research (29). In total, there are 15 primary, 33 secondary, 4 tertiary hospitals in Liangshan.

In total, there are 15 primary, 33 secondary, 4 tertiary hospitals in Liangshan. Using a stratified, random cluster sampling method, we selected three primary, five secondary, and two tertiary hospitals in Liangshan by random number. Our target participants were registered nurses aged 18 or older who had worked in the selected hospitals for at least 6 months and consented to participate.

#### Power Estimate

We calculated the required sample size based on the formula below (30). The required study size was 1,223 (SD = 8.92; effective size δ = 0.50; α = 0.05; Z_α/2_ = 1.96)

$$\begin{array}{l} n={\left(\frac{Z_{\alpha /2}\cdot \text{SD}}{\delta }\right)}^{2}. \end{array}$$

### Data Collection Procedures

In each hospital, we first contacted the head nurse of its nursing department for approval and assistance in recruiting nurses. Once we received approval, we sent eligible nurses an online survey link, including a consent form describing the purpose, procedures, the potential risks and benefits of the study. Nurses who consented to participate received a link to access the online anonymous survey.

### Ethical Considerations

We received approval from the West China Hospital Medical Ethics Committee prior to implementation of the study [No. 430(2017)]. The electronic version of informed consent was received from all participants.

### Variables and Instruments

Our survey consisted of variables shown in the literature to be related to HIV-related stigma in healthcare providers. It included questions about individual and workplace characteristics, HIV-related stigma scale for health care workers (31), and HIV knowledge scale (32). Both the stigma and knowledge scales have been tested in China's population (31, 32).

#### Individual and Workplace Characteristics

Individual characteristics included age, gender, ethnicity, educational level, years of experience working as a nurse, experience of providing care to PLWH, prior experience of HIV-related training, and willingness to receive HIV-related training. Workplace characteristics consisted of questions regarding level of hospital s/he worked, HIV prevalence area (high vs. low; high prevalence was defined as an area with ≥1% HIV prevalence), prior experience working in AIDS specialized hospitals, universal precaution supplies and policies in workplace that protect PLWH from stigma.

#### HIV-Related Stigma

We used a 17-item “HIV-related stigma scale for health care workers” developed by Stein and Li (31) to assess the HIV-related stigma among nurses. This multidimensional scale includes five subscales: Discrimination Intent at Work (four items), Opinion about Health Care for HIV-infected Patients (three items), Prejudiced Attitudes (four items), Internalized Shame (three items), and Fear of PLWHA (three items). Scores for each item ranges from 1 (*strongly agree*) to 5 (*strongly disagree*). All items were reverse-scored when appropriate to have higher total score suggesting higher levels of HIV-related stigma toward PLWH. Stein and Li (31) reported adequate internal consistency (alpha = 0.68–0.82) and construct validity of the scale.

#### HIV Knowledge

We measured HIV knowledge using a Chinese version HIV knowledge scale (32), adapted from Jemmott et al. (33). It is a 24-item scale assessing nurses' knowledge of basic characteristic and transmission routes of HIV. Responses of each scale item were: “*true*,” “*false*” or “*not sure*.” Participants received 1 point if their answers were correct, and they received 0 points if their answers were incorrect or “not sure.” A higher total score indicated higher levels of HIV knowledge.

### Statistical Analyses

We used IBM SPSS 22.0 (34) to manage and analyze data. We used the mean, standard deviation (SD), frequency and percentage to describe distributions of individual and workplace variables, HIV knowledge and HIV-related stigma scores. Pearson's *r* correlation was used to examine correlations between age, years of working experience, HIV knowledge and HIV-related stigma. One-way analyses of variance (ANOVA) for polytomous variables and t-tests for dichotomous variables were used to examine variables that were associated with HIV-related stigma toward PLWH. Variables that were found to be theoretically important and associated with HIV-related stigma at a significant level of *P* < 0.05 at the univariate analysis were entered into the multivariate analysis. We further conducted multiple stepwise regression analysis to investigate associated factors of HIV-related stigma toward PLWH. A *P*-value < 0.05 was used to indicate statistical significance.

## Results

This study recruited 1,289 registered nurses. In total, 9 refused to participate, 22 did not respond and 2 did not complete the questionnaire. Eight of them were excluded because they were younger than 18 years old or had <6 months of work experience. Thus, a final sample of 1,248 participants was included in the analysis.

### Individual and Workplace Characteristics

As shown in Table 1, the mean age of nurses was 30.2 years old (SD = 7.2), with a range of 19–55. Almost all (98.4%) respondents were females; 72.2% (*n* = 902) were Han ethnicity; 62.8% (*n* = 784) had an associated degree; and 90.4% (*n* = 1,128) worked in low prevalence areas. In terms of working experience, the mean year was 8.8 (SD = 7.8). Regarding HIV-related experience and training, although only 12.7% (*n* = 158) of the participants had prior experience working in AIDS specialized hospitals, 84.2% (*n* = 1,051) of them had provided care for PLWH. While 90.1% (*n* = 1,124) of the participants were willing to receive HIV-related training, only 63.1% (*n* = 787) of them had received the training in the past. Majority (95.5%) of the participants reported that the hospitals where they worked provided sufficient universal precaution supplies, and 86.9% (*n* = 1,084) reported that the hospitals had policies to protect PLWH from stigma.

**Table 1:** Individual and workplace characteristics, HIV knowledge and HIV-related stigma toward PLWH.

**Variables**	***N***	**%**	**Mean**	**SD**	**Range**
Age (years)			30.2	7.2	19–55
**Gender**					
Male	20	1.6			
Female	1,228	98.4			
**Ethnicity**					
Han	902	72.2			
Minority	346	27.8			
**Educational level**					
Diploma	92	7.4			
Associate degree	784	62.8			
Baccalaureate degree or above	372	29.8			
Years of working experience			8.8	7.8	0.5-37
**Provide care for PLWH**					
Yes	1051	84.2			
No	197	15.8			
**Willingness to receive HIV-related training**					
Yes	1,124	90.1			
No	124	9.9			
**Prior HIV-related training**					
Yes	787	63.1			
No	461	36.9			
**Level of hospital**					
Primary hospital	102	8.2			
Secondary hospital	592	47.4			
Tertiary hospital	554	44.4			
**HIV prevalence area**					
High prevalence areas (≥1%)	120	9.6			
Low prevalence areas (<1%)	1,128	90.4			
**Prior experience working in AIDS specialized hospitals**					
Yes	158	12.7			
No	1,090	87.3			
**Sufficient universal precaution supplies**					
Yes	1,192	95.5			
No	56	4.5			
**Policies to protect PLWH from stigma**					
Yes	1,084	86.9			
No or doubt	164	13.1			
HIV knowledge			16.3	2.9	7-23
HIV-related stigma scale			50.7	8.3	25-78
Prejudiced attitudes subscale			3.7	0.7	1-5
Fear of PLWHA subscale			3.2	0.8	1-5
Opinion about health care for HIV/AIDS patients subscale			3.1	0.8	1-5
Internalized shame subscale			2.7	0.7	1-5
Discrimination intent at work subscale			2.3	0.6	1-4.8

*PLWH, people living with HIV; SD, standard deviation*.

As for HIV knowledge, the mean total score was 16.3 (SD = 2.9). The mean total score for HIV-related stigma among registered nurses was 50.7 (SD = 8.3). Among the five subscales, “prejudiced attitudes” had the highest scores (M = 3.7, SD = 0.7) while “discrimination intent at work” had the lowest scores (M = 2.3, SD = 0.6).

### Results of Univariate Analysis

The relationships between HIV-related stigma and other study variables are shown in Table 2. A higher HIV-related stigma score was significantly related to longer years of work experience (*r* = 0.06, *P* < 0.05), and lower levels of HIV knowledge(*r* = −0.23, *P* < 0.01). The results of group differences in HIV-related stigma score are presented in Table 3. Nurses who were members of the Han ethnic group (*t* = 3.70, *P* < 0.01), were working in low HIV prevalence areas (*t* = 6.06, *P* < 0.01), had higher educational levels (*F* = 5.89, *P* < 0.05), worked at a higher hospital level (*F* = 11.85, *P* < 0.01), and had not worked in specialized AIDS hospitals (*t* = 4.62, *P* < 0.01) reported higher HIV-related stigma score. Nurses' willingness to receive HIV-related training (*t* = 6.21, *P* < 0.01), experience of having received HIV-related training in the past (*t* = 2.65, *P* < 0.05), and working at a hospital where had policies to protect PLWH from stigma (*t* = 5.70, *P* < 0.01) reported a lower level of HIV-related stigma toward PLWH.

**Table 2:** Correlations between age, years of working experience, HIV knowledge and HIV-related stigma.

**Variables**	**1**	**2**	**3**	**4**
1 Age (years)	1.00	-	-	-
2 Years of working experience	0.95^**^	1.00	-	-
3 HIV knowledge	0.17^**^	0.16^**^	1.00	-
4 HIV-related stigma	0.05	0.06^*^	−0.23^**^	1.00

* *P < 0.05*.

** *P < 0.01*.

**Table 3:** Differences in HIV-related stigma by individual and workplace characteristics^a^.

**Variables**	**Stigma score**		**Statistical test**
	**Mean**	**SD**	
Ethnicity			*t* = 3.70^**^
Han	51.2	8.4	
Minority	49.3	7.9	
Educational level			*F* = 5.89^*^
Diploma	48.0	7.7	
Associate degree	50.7	8.2	
Baccalaureate degree or above	51.3	8.5	
Willingness to receive HIV-related training			*t* = 6.21^**^
Yes	50.2	8.2	
No	55.0	8.1	
Prior HIV-related training			*t* = 2.65^*^
Yes	50.2	8.2	
No	51.5	8.4	
Level of hospital			*F* = 11.85^**^
Primary hospital	48.3	9.8	
Secondary hospital	50.0	8.1	
Tertiary hospital	51.8	8.1	
HIV prevalence area			*t* = 6.06^**^
High prevalence areas	47.0	6.9	
Low prevalence areas	51.1	8.3	
Prior experience working in AIDS specialized hospitals			*t* = 4.62^**^
Yes	47.8	7.4	
No	51.1	8.4	
Policies to protect PLWH from stigma			*t* = −5.70^**^
Yes	50.2	8.2	
No or doubt	54.1	8.1	

*PLWH, people living with HIV; SD, standard deviation*.

a *Only significant results have been listed*.

* *P < 0 .05*.

** *P < 0.01*.

### Findings of Multiple Stepwise Regression Analysis

Table 4 presents the multiple stepwise regression analysis results. The findings suggested that a higher HIV-related stigma was associated with nurses who were more educated (associated degree or higher vs. diploma), had longer years of working experience, were working in the tertiary hospitals, did not have prior experience working in specialized AIDS hospitals, were not willing to receive HIV-related training, worked in low HIV prevalence areas, working in a hospital that had policies to protect PLWH from stigma and had higher levels of HIV knowledge (all *P* < 0.05).

**Table 4:** Multiple stepwise regression analysis of variables in relation to HIV-related stigma.

**Variables**	**B**	**SEE**	**β**	**t**	***P*** **-value**
Educational level	0.95	0.40	0.07	2.37	0.018
Years of working experience (years)	0.10	0.03	0.09	3.33	0.001
**Willingness to receive HIV-related training (ref: yes)**					
No	4.02	0.74	0.15	5.47	<0.001
**Level of hospital (ref: tertiary hospital)**					
Primary hospital	−2.64	0.84	−0.09	−3.15	0.002
Secondary hospital	−1.23	0.48	−0.07	−2.54	0.011
**HIV prevalence area (ref: low prevalence areas)**					
High prevalence areas	−2.56	0.80	−0.09	−3.21	0.001
**Prior experience working in AIDS specialized hospitals (ref: no)**					
Yes	−2.27	0.67	−0.09	−3.40	0.001
**Policy to protect PLWH from stigma (ref: yes)**					
No	3.03	0.65	0.12	4.65	<0.001
HIV knowledge	−0.66	0.08	−0.23	−8.45	<0.001

*SEE, standard error of estimation*.

## Discussion

To the best of our knowledge, this study is the first to exam HIV-related stigma toward PLWH and factors associated with it among nurses in Liangshan, China. The mean score for HIV-related stigma was high, which is similar to findings from research conducted in other regions of China (30, 35). As high levels of HIV-related stigma toward vulnerable PLWH population are related poor nursing care and negative health outcomes (36, 37), PLWH may not receive proper nursing care due to nurses' high levels of HIV-related stigma toward this population in Liangshan.

### Individual Characteristics

Compared with nurses who had an associate degree or higher level of education, diploma-educated nurses reported a lower level of HIV-related stigma, consistent with prior research (38, 39). Deacon and Boulle (40) suggested that higher-educated nurses might have more prejudicial attitudes due to a greater gap in social status between them and PLWH. Similar to other researchers (7, 9, 21), we found that nurses with better HIV knowledge reported no matter what the level of education, a lower level of HIV-related stigma toward PLWH overall, and in particularly, less fear of AIDS. This finding suggested the critical role of HIV knowledge in reducing fear-based stigma (41). Since there was no significant difference of HIV knowledge between nurses with different educational levels, HIV education should target all nurses.

We also found that nurses who had longer years of working experience reported a higher level of HIV-related stigma toward PLWH, a finding similar to that in other studies (17, 22). A possible explanation is that nurses who were more experienced might assign nursing care to nurses with less experience (14). Thus, junior nurses were more likely to provide care to PLWH and this experience might reduce their levels of HIV-related stigma compared with nurses who were more experienced.

### Workplace Characteristics

Consistent with Li et al. (38), we found that nurses working in the tertiary hospitals had a higher level of HIV-related stigma toward PLWH. In China, tertiary hospitals that provide comprehensive services to a larger patient population are more likely to receive referrals of PLWH with opportunistic infections or other illnesses that might not be able to be cured. The complexities of providing care to terminally ill AIDS patients may make these nurses experience burnout and form avoidance attitudes (5, 42).

Our findings supported that nurses working in high HIV prevalence areas and having prior working experience in AIDS specialized hospitals had a lower level of HIV-related stigma, which is also similar to findings in prior research (43, 44). Nurses who worked in high HIV prevalence areas and had prior working experience in AIDS specialized hospitals had more experience in taking care of PLWH. Increasing exposure to and more experience of interacting with PLWH were found to be related to a lower level of HIV-related stigma and higher confidence in taking care of this vulnerable population (23, 45). Moreover, nurses working in high HIV prevalence areas and having prior working experience in AIDS specialized hospitals received more institutional supports and training (43, 44), which were found to reduce their anxiety about HIV infection, thereby reducing their stigma toward PLWH (19, 36, 38).

The significant relationship between nurses' prior HIV-related training and their HIV-related stigma was indicated in *t*-test but not in regression analyses when other variables were held constant. This inconsistent finding may be due to the differences in HIV training, such as content, format and frequency, as they have significant impact on the effectiveness of the training. Further, nurses who were willing to receive HIV-related training reported lower levels of HIV-related stigma. Thus, future research should examine characteristics of successful HIV training for nurses to reduce HIV-related stigma toward PLWH and promote PLWH's optimal health outcomes.

Research has supported the conclusion that stigma-reduction interventions, including policy development, led to positive changes in HIV-related stigma in health care settings (46), which is consistent with our findings. The results indicated that nurses working in the hospitals that implemented policies to protect PLWH from stigma had lower levels of HIV-related stigma toward PLWH. However, we did not find a significant relationship between hospitals that provided sufficient universal precaution supplies and nurses' HIV-related stigma. This might be explained by a lack of variance in this variable, as the majority (95.5%) of nurses reported that universal precaution supplies were provided in the hospitals where they worked.

Given the high prevalence rate of PLWH in Liangshan, providing nurses with comprehensive HIV-related training and requiring hospitals to have policies to reduce HIV-related stigma toward PLWH are critical to reducing the high prevalence rate of PLWH.

## Limitations

Several study limitations should be noted. Given the nature of cross-sectional survey design, it is challenging to draw causal relationships between variables. Further, HIV-related stigma is a sensitive topic in China, and so the validity of self-reported data may not be optimal. With these limitations in mind, our anonymous survey should have minimized the participants' social desirability and bias. However, this online study might exclude people who are not comfortable using technology. Using the stratified random cluster sampling to obtain a powered sample with sufficient sample size enhanced the representation of our target population and therefore increased the external validity of the findings.

## Conclusion

The present study was the first to examine the level of HIV-related sigma toward PLWH and factors associated with the stigma in nurses in Liangshan, an area that has one of the most serious HIV epidemics in China. The findings revealed high levels of HIV-related stigma among nurses in Liangshan. Nurses' high levels of HIV knowledge, willingness to receive HIV-related training, and working in the hospitals that had anti-stigma policies were significantly related to their lower levels of stigma toward PLWH. Comprehensive HIV-related training that addresses these factors and incorporates the unique economic and cultural contexts of Liangshan will help nurses to reduce associated stigma and, in turn, provide culturally congruent care to PLWH and promote their optimal health outcomes.

## Data Availability Statement

The original contributions presented in the study are included in the article/supplementary material, further inquiries can be directed to the corresponding author/s.

## Ethics Statement

We received approval from the West China Hospital Medical Ethics Committee prior to implementation the study [No. 430(2017)]. The participants provided their electronic informed consent to participate in this study.

## Author Contributions

YY, SW, and HC: study design and data collection. YY and AC: analysis and interpretation of data. YY: writing. AC, SW, and HC: critical revisions of manuscript for important intellectual content. SW and HC: supervision of the study. All authors contributed to the article and approved the submitted version.

## Conflict of Interest

The authors declare that the research was conducted in the absence of any commercial or financial relationships that could be construed as a potential conflict of interest.

## Publisher's Note

All claims expressed in this article are solely those of the authors and do not necessarily represent those of their affiliated organizations, or those of the publisher, the editors and the reviewers. Any product that may be evaluated in this article, or claim that may be made by its manufacturer, is not guaranteed or endorsed by the publisher.
